# [4-(Dimethyl­amino)­pyridine-κ*N* ^1^]tri­methyl(thio­cyanato-κ*N*)tin(IV)

**DOI:** 10.1107/S1600536812022064

**Published:** 2012-05-26

**Authors:** Ezzatollah Najafi, Mostafa M. Amini, Seik Weng Ng

**Affiliations:** a Department of Chemistry, General Campus, Shahid Beheshti University, Tehran 1983963113, Iran; b Department of Chemistry, University of Malaya, 50603 Kuala Lumpur, Malaysia; c Chemistry Department, Faculty of Science, King Abdulaziz University, PO Box 80203 Jeddah, Saudi Arabia

## Abstract

In the title monomeric trimethyl­tin(IV) isothio­cyanate–4,4-dimethyl­pyridine adduct, [Sn(CH_3_)_3_(NCS)(C_7_H_10_N_2_)], the Sn^IV^ atom shows a *trans*-C_3_SnN_2_ trigonal bipyramidal coordination. The Sn^IV^ atom lies out of the equatorial plane by 0.033 (4) Å in the direction of the donor N atom of the *N*-heterocycle. The crystal studied was a non-merohedral twin with a minor component of 48.8 (2)%.

## Related literature

For trimethyl­tin isothio­cyanate, see: Forder & Sheldrick (1970 ▶).
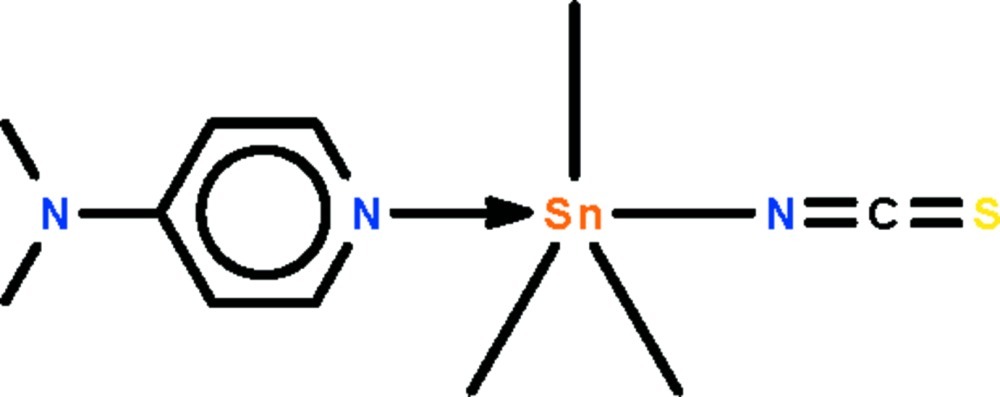


## Experimental

### 

#### Crystal data

[Sn(CH_3_)_3_(NCS)(C_7_H_10_N_2_)]
*M* *_r_* = 344.04
Monoclinic, 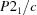

*a* = 7.2026 (4) Å
*b* = 13.4736 (8) Å
*c* = 14.9785 (8) Å
β = 93.792 (5)°
*V* = 1450.41 (14) Å^3^
*Z* = 4
Mo *K*α radiation
μ = 1.89 mm^−1^
*T* = 100 K
0.35 × 0.30 × 0.25 mm

#### Data collection

Agilent SuperNova Dual diffractometer with an Atlas detector
Absorption correction: multi-scan (*CrysAlis PRO*; Agilent, 2012) ▶ *T*_min_ = 0.558, *T*_max_ = 0.650
15682 measured reflections
5542 independent reflections
4916 reflections with *I* > 2σ(*I*)
*R*_int_ = 0.060

#### Refinement

*R*[*F* ^2^ > 2σ(*F* ^2^)] = 0.060
*wR*(*F* ^2^) = 0.197
*S* = 1.23
5542 reflections
151 parameters
H-atom parameters constrained
Δρ_max_ = 1.61 e Å^−3^
Δρ_min_ = −1.98 e Å^−3^

### 

Data collection: *CrysAlis PRO* (Agilent, 2012 ▶); cell refinement: *CrysAlis PRO*; data reduction: *CrysAlis PRO*; program(s) used to solve structure: *SHELXS97* (Sheldrick, 2008 ▶); program(s) used to refine structure: *SHELXL97* (Sheldrick, 2008 ▶); molecular graphics: *X-SEED* (Barbour, 2001 ▶); software used to prepare material for publication: *publCIF* (Westrip, 2010 ▶).

## Supplementary Material

Crystal structure: contains datablock(s) global, I. DOI: 10.1107/S1600536812022064/nk2162sup1.cif

Structure factors: contains datablock(s) I. DOI: 10.1107/S1600536812022064/nk2162Isup2.hkl

Additional supplementary materials:  crystallographic information; 3D view; checkCIF report

## Figures and Tables

**Fig. 1.**
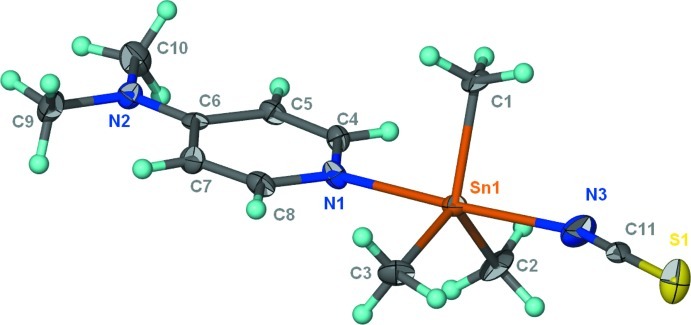
Thermal ellipsoid plot (Barbour, 2001) of (CH3)3Sn(NCS)(C7H10N2) at the 70% probability level; hydrogen atoms are drawn as spheres of arbitrary radius.

## supplementary crystallographic information

### Comment

Trimethyltin halides and pseudohalides are Lewis acids that form 1:1 complexes with aromatic amines. Trimethyltin isocyanate itself exists as a polymer in which the isocyanate anion bridges adjacent trimethyltin cations (Forder & Sheldrick, 1970). In the 4,4-dimethylpyridine adduct (Scheme I), the weaker tin–sulfur bond is disrupted, and the adduct is monomeric. The Sn^IV^ atom shows *trans*-C_3_SnN_2_ trigonal bipyramidal coordination. The tin atom lies out of the equatorial plane by 0.033 (4) Å in the direction of the donor N atom of the *N*-heterocycle.

### Experimental

Trimethyltin isothiocyanate (0.24 g, 1 mmol) and 4-(dimethylamino)pyridine (0.11 g, 1 mmol) were loaded into a convection tube and the tube was filled with methanol and kept at 333 K. Light yellow crystals were collected from the side arm after several days.

### Refinement

Carbon-bound H-atoms were placed in calculated positions [C–H 0.95 to 0.98 Å, *U*_iso_(H) 1.2 to 1.5*U*_eq_(C)] and were included in the refinement in the riding model approximation.

The crystal is a non-merohedral twin having nearly equal components (minor component 48.8 (2) %). A 100% overlap gave the best refinement; however, an artifact of the twinning is the high weighting scheme, which was suggested by the refinement program. The twin law is (-1 0 0 / 0 - 1 0 / 0.2779 0 1).

The final difference Fourier map had a peak 1.08 Å from Sn1 and a hole 0.95 Å from H1a.

### Crystal data

**Table:**

[Sn(CH_3_)_3_(NCS)(C_7_H_10_N_2_)]	*F*(000) = 688
*M**_r_* = 344.04	*D*_x_ = 1.576 Mg m^−^^3^
Monoclinic, *P*2_1_/*c*	Mo *K*α radiation, λ = 0.71073 Å
Hall symbol: -P 2ybc	Cell parameters from 5713 reflections
*a* = 7.2026 (4) Å	θ = 2.7–27.5°
*b* = 13.4736 (8) Å	µ = 1.89 mm^−^^1^
*c* = 14.9785 (8) Å	*T* = 100 K
β = 93.792 (5)°	Prism, light brown
*V* = 1450.41 (14) Å^3^	0.35 × 0.30 × 0.25 mm
*Z* = 4	

### Data collection

**Table:**

Agilent SuperNova Dual diffractometer with an Atlas detector	5542 independent reflections
Radiation source: SuperNova (Mo) X-ray Source	4916 reflections with *I* > 2σ(*I*)
Mirror monochromator	*R*_int_ = 0.060
Detector resolution: 10.4041 pixels mm^-1^	θ_max_ = 27.7°, θ_min_ = 2.7°
ω scan	*h* = −9→9
Absorption correction: multi-scan (*CrysAlis PRO*; Agilent, 2012)	*k* = −17→17
*T*_min_ = 0.558, *T*_max_ = 0.650	*l* = −19→19
15682 measured reflections	

### Refinement

**Table:**

Refinement on *F*^2^	Primary atom site location: structure-invariant direct methods
Least-squares matrix: full	Secondary atom site location: difference Fourier map
*R*[*F*^2^ > 2σ(*F*^2^)] = 0.060	Hydrogen site location: inferred from neighbouring sites
*wR*(*F*^2^) = 0.197	H-atom parameters constrained
*S* = 1.23	*w* = 1/[σ^2^(*F*_o_^2^) + (0.1375*P*)^2^] where *P* = (*F*_o_^2^ + 2*F*_c_^2^)/3
5542 reflections	(Δ/σ)_max_ = 0.001
151 parameters	Δρ_max_ = 1.61 e Å^−^^3^
0 restraints	Δρ_min_ = −1.98 e Å^−^^3^

### Fractional atomic coordinates and isotropic or equivalent isotropic displacement parameters (Å^2^)

**Table:**

	*x*	*y*	*z*	*U*_iso_*/*U*_eq_	
Sn1	0.25215 (5)	0.41625 (3)	0.86069 (2)	0.01334 (16)	
S1	0.3130 (2)	0.35874 (10)	1.18988 (10)	0.0267 (4)	
N1	0.2370 (6)	0.4769 (3)	0.7145 (3)	0.0145 (9)	
N2	0.2613 (7)	0.5696 (3)	0.4491 (3)	0.0160 (10)	
N3	0.2743 (7)	0.3468 (4)	1.0044 (3)	0.0298 (12)	
C1	0.5262 (7)	0.3660 (4)	0.8426 (4)	0.0176 (11)	
H1A	0.6000	0.4208	0.8204	0.026*	
H1B	0.5216	0.3116	0.7991	0.026*	
H1C	0.5835	0.3425	0.8999	0.026*	
C2	0.0214 (8)	0.3222 (5)	0.8288 (4)	0.0247 (13)	
H2A	−0.0670	0.3561	0.7866	0.037*	
H2B	−0.0398	0.3060	0.8835	0.037*	
H2C	0.0644	0.2609	0.8015	0.037*	
C3	0.2037 (8)	0.5581 (4)	0.9169 (4)	0.0219 (12)	
H3A	0.0925	0.5878	0.8865	0.033*	
H3B	0.3113	0.6011	0.9092	0.033*	
H3C	0.1851	0.5509	0.9807	0.033*	
C4	0.2356 (7)	0.4114 (4)	0.6457 (3)	0.0146 (11)	
H4	0.2297	0.3427	0.6592	0.018*	
C5	0.2421 (8)	0.4383 (4)	0.5584 (4)	0.0151 (11)	
H5	0.2408	0.3884	0.5135	0.018*	
C6	0.2507 (7)	0.5397 (3)	0.5334 (3)	0.0118 (10)	
C7	0.2515 (8)	0.6078 (4)	0.6063 (4)	0.0159 (11)	
H7	0.2566	0.6771	0.5952	0.019*	
C8	0.2448 (8)	0.5743 (3)	0.6913 (4)	0.0152 (11)	
H8	0.2457	0.6221	0.7379	0.018*	
C9	0.2689 (9)	0.6754 (4)	0.4273 (4)	0.0232 (12)	
H9A	0.3785	0.7053	0.4590	0.035*	
H9B	0.1563	0.7083	0.4458	0.035*	
H9C	0.2771	0.6834	0.3627	0.035*	
C10	0.2531 (8)	0.4977 (5)	0.3755 (4)	0.0224 (12)	
H10A	0.3642	0.4556	0.3805	0.034*	
H10B	0.2477	0.5331	0.3183	0.034*	
H10C	0.1418	0.4563	0.3786	0.034*	
C11	0.2906 (7)	0.3530 (4)	1.0821 (4)	0.0171 (11)	

### Atomic displacement parameters (Å^2^)

**Table:**

	*U*^11^	*U*^22^	*U*^33^	*U*^12^	*U*^13^	*U*^23^
Sn1	0.0109 (2)	0.0157 (2)	0.0135 (2)	0.00108 (13)	0.00189 (17)	0.00061 (12)
S1	0.0435 (9)	0.0206 (7)	0.0158 (7)	−0.0071 (7)	−0.0002 (7)	−0.0019 (5)
N1	0.017 (2)	0.0109 (19)	0.015 (2)	0.0021 (17)	−0.0028 (19)	−0.0013 (16)
N2	0.020 (3)	0.014 (2)	0.014 (2)	−0.0016 (18)	0.001 (2)	0.0002 (16)
N3	0.031 (3)	0.042 (3)	0.017 (3)	0.004 (3)	0.009 (2)	0.003 (2)
C1	0.004 (2)	0.028 (3)	0.020 (3)	−0.003 (2)	−0.001 (2)	0.006 (2)
C2	0.014 (3)	0.030 (3)	0.030 (3)	−0.015 (2)	0.003 (2)	0.004 (3)
C3	0.021 (3)	0.024 (3)	0.022 (3)	0.002 (2)	0.008 (3)	−0.006 (2)
C4	0.016 (3)	0.011 (2)	0.017 (3)	0.0000 (18)	0.000 (3)	0.0000 (18)
C5	0.015 (3)	0.013 (2)	0.017 (3)	0.002 (2)	0.001 (2)	−0.0027 (19)
C6	0.006 (2)	0.013 (2)	0.016 (2)	−0.0007 (18)	0.001 (2)	−0.0023 (19)
C7	0.016 (3)	0.012 (2)	0.019 (3)	−0.003 (2)	0.002 (2)	0.000 (2)
C8	0.015 (3)	0.012 (2)	0.019 (3)	0.0017 (19)	0.000 (2)	−0.0031 (18)
C9	0.032 (3)	0.020 (3)	0.019 (3)	−0.003 (2)	0.008 (3)	0.004 (2)
C10	0.026 (3)	0.027 (3)	0.013 (2)	0.004 (2)	0.000 (2)	−0.001 (2)
C11	0.007 (2)	0.018 (2)	0.027 (3)	0.000 (2)	0.004 (2)	0.008 (2)

### Geometric parameters (Å, º)

**Table:**

Sn1—C2	2.120 (5)	C3—H3A	0.9800
Sn1—C1	2.121 (5)	C3—H3B	0.9800
Sn1—C3	2.126 (5)	C3—H3C	0.9800
Sn1—N1	2.333 (4)	C4—C5	1.360 (7)
Sn1—N3	2.344 (5)	C4—H4	0.9500
S1—C11	1.614 (6)	C5—C6	1.419 (6)
N1—C4	1.357 (6)	C5—H5	0.9500
N1—C8	1.359 (6)	C6—C7	1.426 (7)
N2—C6	1.333 (6)	C7—C8	1.354 (8)
N2—C9	1.464 (6)	C7—H7	0.9500
N2—C10	1.466 (7)	C8—H8	0.9500
N3—C11	1.164 (7)	C9—H9A	0.9800
C1—H1A	0.9800	C9—H9B	0.9800
C1—H1B	0.9800	C9—H9C	0.9800
C1—H1C	0.9800	C10—H10A	0.9800
C2—H2A	0.9800	C10—H10B	0.9800
C2—H2B	0.9800	C10—H10C	0.9800
C2—H2C	0.9800		
			
C2—Sn1—C1	120.2 (2)	Sn1—C3—H3C	109.5
C2—Sn1—C3	118.7 (2)	H3A—C3—H3C	109.5
C1—Sn1—C3	121.1 (2)	H3B—C3—H3C	109.5
C2—Sn1—N1	90.6 (2)	N1—C4—C5	124.0 (4)
C1—Sn1—N1	88.74 (18)	N1—C4—H4	118.0
C3—Sn1—N1	93.30 (18)	C5—C4—H4	118.0
C2—Sn1—N3	88.5 (2)	C4—C5—C6	120.9 (5)
C1—Sn1—N3	89.0 (2)	C4—C5—H5	119.5
C3—Sn1—N3	89.9 (2)	C6—C5—H5	119.5
N1—Sn1—N3	176.70 (16)	N2—C6—C5	123.2 (4)
C4—N1—C8	115.5 (5)	N2—C6—C7	122.2 (5)
C4—N1—Sn1	118.9 (3)	C5—C6—C7	114.6 (5)
C8—N1—Sn1	125.4 (3)	C8—C7—C6	120.4 (5)
C6—N2—C9	120.7 (4)	C8—C7—H7	119.8
C6—N2—C10	120.7 (4)	C6—C7—H7	119.8
C9—N2—C10	118.4 (5)	C7—C8—N1	124.6 (5)
C11—N3—Sn1	152.3 (5)	C7—C8—H8	117.7
Sn1—C1—H1A	109.5	N1—C8—H8	117.7
Sn1—C1—H1B	109.5	N2—C9—H9A	109.5
H1A—C1—H1B	109.5	N2—C9—H9B	109.5
Sn1—C1—H1C	109.5	H9A—C9—H9B	109.5
H1A—C1—H1C	109.5	N2—C9—H9C	109.5
H1B—C1—H1C	109.5	H9A—C9—H9C	109.5
Sn1—C2—H2A	109.5	H9B—C9—H9C	109.5
Sn1—C2—H2B	109.5	N2—C10—H10A	109.5
H2A—C2—H2B	109.5	N2—C10—H10B	109.5
Sn1—C2—H2C	109.5	H10A—C10—H10B	109.5
H2A—C2—H2C	109.5	N2—C10—H10C	109.5
H2B—C2—H2C	109.5	H10A—C10—H10C	109.5
Sn1—C3—H3A	109.5	H10B—C10—H10C	109.5
Sn1—C3—H3B	109.5	N3—C11—S1	178.7 (5)
H3A—C3—H3B	109.5		
			
C2—Sn1—N1—C4	−52.6 (4)	C9—N2—C6—C5	179.7 (5)
C1—Sn1—N1—C4	67.6 (4)	C10—N2—C6—C5	3.8 (8)
C3—Sn1—N1—C4	−171.4 (4)	C9—N2—C6—C7	−1.8 (8)
C2—Sn1—N1—C8	133.2 (5)	C10—N2—C6—C7	−177.7 (5)
C1—Sn1—N1—C8	−106.6 (5)	C4—C5—C6—N2	178.5 (5)
C3—Sn1—N1—C8	14.4 (5)	C4—C5—C6—C7	−0.1 (8)
C2—Sn1—N3—C11	−133.1 (10)	N2—C6—C7—C8	−178.4 (5)
C1—Sn1—N3—C11	106.7 (10)	C5—C6—C7—C8	0.2 (8)
C3—Sn1—N3—C11	−14.4 (10)	C6—C7—C8—N1	−0.1 (9)
C8—N1—C4—C5	0.3 (8)	C4—N1—C8—C7	−0.2 (8)
Sn1—N1—C4—C5	−174.5 (4)	Sn1—N1—C8—C7	174.2 (5)
N1—C4—C5—C6	−0.1 (9)		

## References

[bb1] Agilent (2012). *CrysAlis PRO* Agilent Technologies, Yarnton, England.

[bb2] Barbour, L. J. (2001). *J. Supramol. Chem.* **1**, 189–191.

[bb3] Forder, R. A. & Sheldrick, G. M. (1970). *J. Organomet. Chem.* **21**, 115–122.

[bb4] Sheldrick, G. M. (2008). *Acta Cryst.* A**64**, 112–122. 10.1107/S0108767307043930 18156677

[bb5] Westrip, S. P. (2010). *J. Appl. Cryst.* **43**, 920–925.

